# Assessing Clinical Outcomes of Metronidazole for Intra-Abdominal Infections When Dosed Every 12 h Versus Every 8 h in a Multi-Center Health System

**DOI:** 10.3390/antibiotics14070688

**Published:** 2025-07-08

**Authors:** Sarah Galante, Ramya Castillo, Todd Price, MaiCuc Tran, Stefanie Stramel-Stafford

**Affiliations:** Memorial Hermann Memorial City Medical Center, Houston, TX 77024, USA

**Keywords:** metronidazole, intra-abdominal, pharmacokinetic, dosing, infectious diseases, anaerobic

## Abstract

**Background**: Metronidazole is the preferred anaerobic agent for empiric treatment of intra-abdominal infections (IAI). Although dosed every 8 h (q8hr), blood concentrations exceed the in vitro minimum inhibitory concentration (MIC) for anaerobic organisms at 12 h (q12hr). A drug shortage of intravenous (IV) metronidazole prompted the conversion to every 12 h dosing in qualifying patients treated for IAI. **Objective**: To determine efficacy outcomes of metronidazole dosed every 12 h versus every 8 h in patients treated for IAI. **Methods**: This was a multi-center, retrospective, cohort study of 201 patients from January to July 2021 (q8hr) and January to November 2023 (q12hr) at five hospitals through the greater Houston area. Included patients were adults with a diagnosis of IAI confirmed by radiographic evidence and a white blood count (WBC) > 12,000 cells/µL and/or temperature > 100.4 °F at the time of diagnosis. The primary outcome was clinical cure of IAI, defined as resolution of signs/symptoms of IAI and normalization of WBC or temperature. **Results**: A total of 201 patients were included, 103 patients in the q8hr group and 98 patients in the q12hr group. Clinical cure of IAI occurred in 72 patients (69.9%) in the q8hr group and 62 patients (63.2%) in the q12hr group (*p* = 0.318). The median duration of therapy days was similar for both groups (4.0 [4.0–6.0] vs. 4.0 [3.0–6.0] (*p* = 0.509)). The frequency of clinical failure was higher in the q12hr group (8.7% vs. 21.4%; *p* = 0.01). Seven patients in the q8hr group and fourteen patients in the q12hr group required escalation of antibiotics due to the need for broader-spectrum antimicrobial therapy by clinical failure definition. **Conclusions**: There was no difference in clinical cure of IAI with an extended dosing interval. Clinical failure and escalation in antibiotics was higher in the q12h group due to the need for broader-spectrum gram-negative coverage and not related to the need for anaerobic coverage. Findings suggest that every 12 h dosing has similar outcomes.

## 1. Introduction

Metronidazole is considered the drug of choice for the treatment of anaerobic infections [1,2,3]. It is unique compared to other antibiotics in that the spectrum of activity is limited to both gram-negative and gram-positive anaerobic organisms. These organisms include, but are not restricted to, *Bacteroides fragilis*, *Prevotella* sp., *Fusobacterium* sp., *Gardnerella vaginalis*, *Clostridium* sp., and *Peptostreptococcus* sp. [1,4]. Anaerobic organisms are commonly present in the oral and gastrointestinal spaces as a part of the normal human flora [4,5]. When infection occurs at these sites, anaerobes should be considered among the causative pathogens. The 2017 Surgical Infection Society Revised Guidelines on the Management of IAI recommends metronidazole as a preferred antibiotic for empiric treatment [2]. IAI involves organs and spaces within the gastrointestinal tract and abdominal cavity and encompasses infection types such as diverticulitis, appendicitis, cholangitis, cholecystitis, abscesses, and peritonitis. Anaerobes are usually difficult to identify via culture due to slow growth and inability to maintain an anerobic environment during cultivation; therefore, empiric anaerobic coverage is often utilized [6]. IAIs are typically polymicrobial in nature; thus, metronidazole is frequently used in combination with other antibiotics for broad-spectrum coverage.

Resistance rates to metronidazole are low, it is available in both oral and intravenous (IV) formulations, it penetrates well into the body tissues, and it is well tolerated [1,4,7]. It is generally dosed every 8 h; however, there is pharmacokinetic data to support that blood concentrations exceed the minimum inhibitory concentration (MIC) for anaerobic organisms at 12 h [8,9,10]. The half-life of metronidazole is approximately 8 h and it has a post antibiotic effect of about 3 h, which adds to the justification for a q12hr dosing strategy [8]. There is also emerging clinical data to support the role of q12hr dosing in practice. A retrospective study published by Soule and colleagues in 2018 [11] evaluated q12hr metronidazole dosing in patients with presumed anaerobic or mixed anaerobic infections. The authors found no differences in clinical cure, duration of antibiotics, hospital length of stay, escalation of antibiotic therapy, microbiologic cure, or mortality between q8hr and q12hr dosing of metronidazole. Of note, 84% of patients included in this study received metronidazole for treatment of IAI [11].

In the fall of 2021, there was a national drug shortage of IV metronidazole. Given the historic pharmacokinetic data to support q12hr dosing, there is utility in further evaluating this dosing strategy to add to the emerging clinical data and the impact on clinical outcomes. This prompted the implementation of a drug shortage action plan as a conservation strategy. The pharmacist-driven action plan prompted pharmacists to automatically convert patients on IV therapy to oral (PO) metronidazole if they met the system criteria for IV to PO conversion and automatically convert patients receiving q8hr dosing to q12hr dosing. The protocol excluded patients < 18 years of age or those being treated for *Clostridioides difficile*, central nervous system infection, parasitic/amoebic infection, surgical prophylaxis, or *Helicobacter pylori* [11].

The objective of this study was to determine if metronidazole dosed q12hr has similar efficacy compared to q8hr dosing in patients treated for IAI. The primary outcome was clinical cure of IAI, defined as resolution of signs/symptoms of IAI and normalization of WBC and/or temperature, by end of therapy or discharge, whichever presented first. Secondary outcomes included clinical failure, duration of therapy, hospital length of stay, and readmission within 30 days for IAI.

## 2. Results

A total of 201 patients were included in this study, with 103 patients in the q8hr dosing group and 98 patients in the q12hr dosing group. Baseline characteristics were similar between the two groups (Table 1). The average age was 58.5 ± 19.3 years in the q8hr group and 60.6 ± 19.5 years in the q12hr group (*p* = 0.431). The most common diagnosis types between the two groups were diverticulitis (23.3% vs. 15.3%), cholecystitis (15.5% vs. 12.2%), and IAI abscess (11.7 vs. 17.3), which were generally associated with another infection type (e.g., diverticulitis). Most patients in each group did not have a surgical intervention; therefore, complete source control may not have been achieved. Additionally, all patients included in this study received concurrent therapy with at least one other antibiotic in addition to metronidazole, due to the polymicrobial nature of IAI.

Primary and secondary outcomes are reported in Table 2. There was no difference in clinical cure of IAI, which occurred in 69.9% of patients in the q8hr group and 63.2% of patients in the q12hr group (*p* = 0.318). The frequency of clinical failure was higher in the q12hr group (8.7% vs. 21.4%; *p* = 0.01). Seven patients in the q8hr group and fourteen patients in the q12hr group required escalation of antibiotics due to the need for broader-spectrum antimicrobial therapy by clinical failure definition. All patients required escalation to a carbapenem or piperacillin/tazobactam. Two patients in the q8hr group and three patients in the q12hr group required escalation of inpatient status (transfer to intermediate care unit or intensive care unit). The median duration of therapy for metronidazole specifically was 4 days for both groups ([4.0–6.0] vs. [3.0–6.0]; *p* = 0.549). The median length of hospital stay was similar between the groups; however, it was slightly longer for patients in the q12hr group (4.2 days [3.0–6.7] vs. 4.9 days [3.5–8.6]; *p* = 0.026). Readmission in 30 days for IAI was 8.7% in the q8hr group and 14.2% in the q12hr group, but was not significantly different (*p* = 0.217).

## 3. Discussion

This multi-center, retrospective, pre–post-intervention study found no difference in clinical cure of IAI between q8hr and q12hr dosing of metronidazole. There was also no difference in the secondary outcomes of duration of therapy and readmission within 30 days for IAI. These findings are consistent with existing pharmacokinetic data and limited clinical data.

Failure of metronidazole therapy was primarily due to escalation of antibiotics and worsening symptoms of IAI. In the pre-intervention group, patients who required escalation of antibiotics only had one patient with worsening symptoms after 48 h of metronidazole initiation, whereas, in the post-intervention group, only two patients had worsening symptoms after 48 h of metronidazole initiation. Those with infection caused by extended-spectrum beta-lactamase (ESBL) *E. coli* required antibiotic escalation to a susceptible agent. Furthermore, worsening symptoms may have been attributed to the non-anerobic organism, as improvement would be expected after metronidazole initiation for anaerobic infections. Symptoms of IAI assessed included abdominal pain, nausea, vomiting, or diarrhea. Metronidazole is generally well tolerated, although gastrointestinal effects are a known side effect [1]. One patient in the post-intervention group was escalated to meropenem due to intolerance. Therefore, it is uncertain if worsening symptoms for other patients was solely due to infection. Decreasing the metronidazole dosing frequency to q12hr reduces the overall exposure to the drug, which may ultimately reduce the risk of adverse effects.

Hospital length of stay was similar as both were about 4 days between the two groups, with 4.2 vs. 4.9 median days; however, it was statistically 1 day longer in the q12hr dosing group (4.2 days [3.0–6.7] vs. 4.9 days [3.5–8.6]; *p* = 0.026). The increased length of hospital stay was likely affected by one patient who experienced complications that required additional surgery to obtain source control. Additionally, multiple patients in the q12hr group had lengths of stay greater than 20 days due to additional comorbidities and medical conditions other than IAI requiring prolonged treatment. Only one patient in the q8hr group with escalated therapy had a length of stay of over 20 days. There was no difference in readmission within 30 days for IAI, suggesting that q12hr dosing has similar efficacy and safety compared to q8hr dosing. Two patients in the q12hr group were specifically noted to have readmissions not related to IAI.

A strength of this study is that it is the first to evaluate dosing metronidazole q8hr vs. q12hr specifically for treatment of IAI. IAI is one of the most common indications for metronidazole as it is often utilized for empiric treatment. The study by Soule et al. in 2018 [11] evaluated anaerobic or mixed anaerobic infections for a variety of infection types and, notably, the most common infection type was abdominal infection. Additionally, a narrower efficacy margin of 15% was utilized in this study to calculate the sample size, which is stricter than the typical 20% used in the previous study [11]. Another strength is that it is a muti-center study, including various hospitals in the Greater Houston area with a wide range of bed sizes, services offered, acuity (including fluctuations), and patient populations. This allows for greater generalizability of results. This study does have several limitations. First, most patients were on concomitant antibiotic therapy in addition to metronidazole and anerobic pathogens were rarely identified. This makes it difficult to determine if metronidazole treatment was the primary reason for clinical cure; however, most patients with IAI are often treated with combination therapy. Also, since microbiological results were not available, it is not possible to clearly establish the reasons for the clinical failures in our patients. Anaerobic bacteria are not usually identified in practice as they are difficult to isolate, and anaerobic cultures are not usually recommended by current guidelines for patients with community-acquired IAI [2,3]. Second, this study was restricted to only 5 out of 12 sites within the healthcare system. Increasing the number of sites would assist in capturing more patients for inclusion. Lastly, as q8hr dosing is still recommended by most guidelines, a prospective study would be of benefit to help address future studies.

In this study, there was no difference in clinical cure between metronidazole dosed q8hr vs. q12hr in patients treated for IAI. These findings support historical pharmacokinetic data and emerging clinical data evaluating these dosing frequencies [8,9,10,11]. Dosing metronidazole q12hr may be beneficial for increasing patient compliance upon discharge, reducing adverse effects, and ultimately reducing antibiotic resistance due to less frequent use.

## 4. Materials and Methods

This was a multi-center, retrospective, pre–post-intervention study of 201 patients with IAIs at 5 Memorial Hermann Health System locations between January 2021 and November 2023. The study locations include Memorial City, Texas Medical Center, The Woodlands, Greater Heights, and Cypress, located in the greater Houston, Texas, USA, area. These hospitals included 1 large academic medical center and 4 community hospitals, all with bed sizes ranging from approximately 100 to over 1000. The pre-intervention group received metronidazole q8hr between January 2021 and July 2021. In the fall of 2021, a drug shortage action plan was implemented as a conservation strategy; therefore, the post-intervention group received metronidazole q12hr between January 2022 and November 2023. This study was approved by the University of Texas Health Science Center at Houston Institutional Review Board.

### 4.1. Patients

Patients were initially identified if they received metronidazole as inpatients between January and July of 2021 or January and November 2023 using a third-party data collection tool. Patients were included if they were at least 18 years of age, received metronidazole for ≥48 h for treatment of IAI, had confirmed IAI via radiographic evidence (computed tomography scan or ultrasound), and had an elevated WBC > 12,000 cells/µL and/or temperature >100.4 °F. Patients were excluded if they received neither q8hr or q12hr dosing, ≥1 dose of metronidazole q8hr before transitioning to q12hr or vice versa, ≥48 h of an anaerobic agent other than metronidazole, metronidazole prior to admission, or if they were pregnant or being treated for *Clostridioides difficile*, central nervous system infection, parasitic/amoebic infection, surgical prophylaxis, or *Heliobacter pylori*. Informed consent was not obtained as this was a retrospective study.

### 4.2. Definitions

Clinical cure was defined as resolution or reduction in signs and symptoms of IAI, normalization of WBC count (4000 to 12,000 cells/µL), and/or temperature (>96.8 °F and <100.4 °F) by the end of therapy or discharge, whichever presented first. Normalization of WBC and temperature consisted of two consecutive values at least 8 h apart. Clinical failure was defined as escalation of antibiotic therapy, worsening symptoms after 48 h of metronidazole therapy, or escalation of inpatient status due to worsening infection. If a patient met criteria for clinical failure, then they were no longer eligible to be assessed for clinical cure. Escalation of antibiotics was defined as transitioning from metronidazole to a broad-spectrum agent with anaerobic coverage. Readmission in 30 days accounted for patients who were readmitted specifically for treatment of IAI.

### 4.3. Statistical Analysis

A total of 98 patients were needed in each group to meet a power of 0.80 and detect a 15% difference in clinical cure rates, assuming an 85% response rate in both groups and an α of 0.05 [11]. Clinical cure, clinical failure, and readmission within 30 days were analyzed using a Chi-square test. Duration of therapy and length of stay were analyzed using a Mann–Whitney U test. A *p* value of 0.05 determined statistical significance.

## Figures and Tables

**Table 1:** Baseline characteristics.

	q8hr (*n* = 103)	q12hr (*n* = 98)	*p*-Value
Age in years, mean ± SD	58.5 ± 19.3	60.6 ± 19.5	0.431
Male, *n* (%)	51 (49.5)	41 (41.8)	0.275
BMI, mean ± SD	29.2 ± 7.9	28.2 ± 7.5	0.373
Diagnosis, *n* (%)			
Appendicitis	13 (12.6)	4 (4.1)	
Diverticulitis	24 (23.3)	15 (15.3)	
Cholecystitis	16 (15.5)	12 (12.2)	
IAI abscess	12 (11.7)	17 (17.3)	
Other	38 (36.9)	50 (51.0)	

**Table 2:** Outcomes.

	q8hr (*n* = 103)	q12hr (*n* = 98)	*p*-Value
Clinical cure of IAI, *n* (%)	72 (69.9)	62 (63.2)	0.318
Clinical failure, *n* (%)	9 (8.7)	21 (21.4)	0.01
Escalation of antibiotics, *n* (%)	7 (6.8)	14 (14.3)	
Worsening symptoms, *n* (%)	1 (1.0)	2 (2.0)	
Escalation of inpatient status, *n* (%)	2 (1.9)	3 (3.1)	
Duration of therapy in days, median (IQR)	4.0 (4.0–6.0)	4.0 (3.0–6.0)	0.549
Length of hospital stay in days, median (IQR)	4.2 (3.0–6.7)	4.9 (3.5–8.6)	0.026
Readmission in 30 days for IAI, *n* (%)	9 (8.7)	14 (14.2)	0.217

## Data Availability

Data is unavailable due to privacy or ethical restrictions.
